# Supplementary material for: Evaluating the Chemical Composition and Antitumor Activity of Origanum vulgare ssp. hirtum Essential Oil in a Preclinical Colon Cancer Model
Source: Int J Mol Sci. 2025 May 15;26(10):4737. doi: 10.3390/ijms26104737 (PMC12111866; doi:10.3390/ijms26104737)
Supplement: Supplementary file 1 [file ijms-26-04737-s001.zip › ijms-3607578-supplementary.pdf]

Supplementary Methods

Cell isolation from tumors and lymph nodes

For the analysis of immune cell infiltration, excised tumors were washed with PBS and minced in ~ 1 mm pieces. These were then incubated with 0.5 mg/ml collagenase IV in RPMI medium for 1 h at 37°C under shaking. Digested tissue was filtered through a 100-μm strainer to acquire a single-cell suspension and cells were washed twice with PBS prior to staining. Lymph node cells were isolated by mechanically homogenizing the tissue through the strainer, pelleting the cells by centrifugation at 4°C, 500 × g for 5 minutes and washing the cells twice with PBS prior to staining.

Flow cytometry

Immunophenotype analysis of immune cells was examined with flow cytometry. Isolated cells were incubated with antibodies conjugated with fluorescent dyes at 4°C for 40 minutes in FACS buffer (PBS, 2.5% FBS, 2.5 mM EDTA). After staining cells were washed twice with FACS buffer and resuspended in PBS. An Attune NxT flow cytometer was used for data collection and FlowJo software for the analysis.

Origanum vulgare ssp. hirtum essential oil toxicity assessment

In order for the potential toxicity of the essential oil to be examined, female BALB/c mice, not injected with cancer cells, were orally administered 0.348 mg/kg of bw or 2.61 mg/kg of bw for 3 or 10 days, while control mice received only corn oil (n = 3 for all groups). Blood was collected at the end of the administration period and levels of liver enzymes (SGOT, SGPT and ALP) were measured. Mice were also monitored throughout the experiment for signs of pain, discomfort or weight loss.

Supplementary Tables

Table S1: The limit of detection (LoD), quantification (LoQ), linearity, precision, and accuracy results for the screened polyphenolic compounds contained in *Origanum vulgare spp hirtum.* The calibration equations represent the peak area as a function of concentration in ppb. The intra- and inter-day experimental data concern data that have been collected from a five-day experiment, whereas the %recovery data are the means of three independent experiments.

Compound	Linear range (ppb)	LOD (ppb)	LOQ (ppb)	Calibration equation[a]	Correlation coefficient (r^2)	%RSD		%REC[d]
						(intra-day)[b]	(inter-day)[c]	
**Benzoic acid derivatives**								
m-hydroxybenzoic acid	13.39-499.21	13.39	40.58	y=42.16x+277.25	0.9997	1.00	2.06	99.9
Protocatechuic acid	0.66-504.50	0.66	14.70	y=34.24x-69.4	0.9995	1.25	2.65	86.3
Vanillin	2.87-335.00	2.87	5.62	y=0.67x-0.1	0.9999	0.98	0.95	100.4
p-hydroxy benzaldehyde	0.57-99.46	0.57	1.72	y=7.43x+36.81	0.9991	3.21	3.25.	95.6
**Gallic acid derivatives**								
Gallic acid	53.20-513.20	53.20	105.20	y=0.67x-1.5	0.9996	0.46	0.21	99.9
Ethyl gallate	1.21-108.36	1.21	3.69	y=21.07x+123.35	0.9999	1.06	1.33	97.5
**Cinnamic acid derivatives**								
Ferulic acid	2.10-505.60	2.10	12. 17	y=19.02x-68.4	0.9992	0.7	2.45	102.6
Caffeic acid	1.21-500	1.21	1.25	y=92.95x+344.4	0.9995	1.01	2.21	100.1
Dihydrocaffeic acid	97.28-499.66	97.28	294.81	y=11.92+64.06	0.9998	0.97	1.14	94.4
p-coumaric acid	0.65-497.30	0.65	1.55	y=52.84x+36.9	0.9997	1.7	1.94	93.2
Chlorogenic acid	3.48-495.60	3.48	4.76	y=25.02x+60.3	0.9991	1.35	1.98	87.4
**Coumarin derivatives**								
Coumarin	0.91-497.55	0.91	2.76	y=2370.35x+15986.5	0.9997	2.36	3.62	92.1
m-hydroxycoumarin	11.84-503.71	11.84	35.90	y=2.59x+22.96	0.9970	1.34	3.01	97.8
**Phenolic derivative**								

Eugenol	3.63-497.40	3.63	11.01	y=89.12x+643	0.9987	2.25	4.68	101.2
**Furanocoumarin derivatives**								
Xanthotoxol	0.27-498.16	0.27	0.81	y=1276.71x+9756.89	0.9992	1.58	2.21	95.5
**Flavanone derivatives**								
4'-methoxyflavanone	2.21-250.00	2.21	3.89	y=83.54x+60.3	0.9999	2.89	1.87	93.6
Naringin	3.01-250.60	3.01	1.21	y=22.88x-43.3	0.9997	2.22	4.02	95.4
**Flavonol derivatives**								
Isorhamnetin	14.01-251.1	14.01	2.31	y=6.08x-15.4	0.9992	2.48	1.18	100.1
Quercetin-3-*O*-rhamnoside	1.02-250.60	1.02	4.21	y=60.83x-38.6	0.9999	2.21	3.01	99.8
Myricetin-3-*O*-rhamnoside	3.12-500.00	3.12	9.47	y=17.99x+80.65	0.9997	1.10	2.01	100.6
Myricetin-3-*O*-galactoside	0.85-251.20	0.85	2.12	y=26.38x-31.8	0.9997	1.78	1.65	100.2
Kaempferol	10.61-499.28	10.61	32.16	y=2.59543x+14.47	0.9995	2.32	2.69	89.9
**Procyanidin**								
Procyanidin-B2	2.04-498.94	2.04	6.18	y=10.76x+42.68	0.9998	2.36	3.12	96.3

Table S2: The optimal conditions for Multiple Reaction Monitoring (MRM) transitions of phenolic acid and flavonoids in *Origanum vulgare spp hirtum*.

Polyphenolic compound	Chemical formula	Molecular weight	[M-H]± (m/z)	ESI (±)	MS² fragments (m/z)	Cone voltage (V)	Collision energy (eV)	Retention time (R_t)
**Benzoic acid derivatives**								
m-hydroxy benzoic acid	$C_7H_6O_3$	138.13	137.05	-	92.9	22	10	2.34
Protocatechuic acid	$C_7H_6O_4$	154.12	152.95	-	108.95	25	13	1.64
Vanillin	$C_8H_8O_3$	152.15	151.0	-	92.2<br>136.0	22	20<br>15	2.23
p-hydroxy benzaldehyde	$C_7H_6O_2$	122.13	120.95	-	91.85	12	20	4.69
**Gallic acid derivatives**								
Gallic acid	$C_7H_6O_5$	170.12	168.95	-	78.98<br>124.95	23	22<br>15	1.37
Ethyl gallate	$C_9H_{10}O_5$	198.18	197.05	-	124.0	15	25	4.66
**Cinnamic acid derivatives**								
Ferulic acid	$C_{10}H_{10}O_4$	194.18	192.95	-	134.0<br>178.0	26	25<br>12	2.20
Caffeic acid	$C_9H_8O_4$	180.16	178.95	-	134.95	25	13	1.89
Dihydro caffeic acid	$C_9H_{10}O_4$	182.17	181.05	-	137.05	22	12	4.39
Chlorogenic acid	$C_{16}H_{18}O_9$	354.31	353.1	-	84.0<br>191.02	22	44<br>14	1.70
**Coumarin derivatives**								
Coumarin	$C_9H_6O_2$	146.15	147.01	+	91<br>102.9	17	12<br>17	5.44
m-hydroxy coumarin	$C_9H_6O_3$	162.15	161.15	-	133	17	13	5.26
**Phenolic derivative**								
Eugenol	$C_{10}H_{12}O_2$	164.21	165.25	+	123.95	14	15	6.08
**Furanocoumarin derivatives**								
Xanthotoxol	$C_{11}H_6O_4$	202.17	203.15	+	131.15<br>147.05	22	18<br>21	5.09
**Flavanones derivatives**								
4'-methoxyflavanone	$C_{16}H_{14}O_3$	254.29	255.15	+	240<br>161.3	31	17<br>22	3.78
Naringin	$C_{22}H_{32}O_{14}$	580.54	579.15	-	271.1<br>151.5	45	33<br>40	2.21

**Flavonol derivatives**								
Isorhamnetin	$C_{16}H_{12}O_7$	316.28	315	-	151.0<br>300.2	43	30<br>20	2.86
Quercetin-3-*O*-rhamnoside	$C_{21}H_{20}O_{11}$	448.38	447.01	-	271.0<br>300.0	43	47<br>28	2.14
Myricetin-3-galactoside	$C_{21}H_{20}O_{13}$	480.38	479.05	-	271.1<br>287.1	48	39<br>44	1.87
Myricetin-3-O-rhamnoside	$C_{21}H_{20}O_{12}$	464.38	463.15	-	316.3<br>271.25	31	27<br>42	4.38
Kaempferol	$C_{15}H_{10}O_6$	286.25	285.25	-	151	30	14	5.32
**Procyanidin**								
Procyanidin-B2	$C_{30}H_{26}O_{12}$	578.53	577	-	125.05<br>289.3<br>407.35	29	32<br>23<br>24	2.30

Table S3: Real-time qPCR primers.

	Forward 5 → 3	Reverse 5 → 3
beta actin	GGCTGTATTCCCCTCCATCG	CCAGTTGGTAACAATGCCATGT
GAPDH	CATCACTGCCACCCAGAAGACTG	ATGCCAGTGAGCTTCCCGTTCAG
IFNa2	AGCAGATCCAGAAGGCTCAA	GGAGGGTTGTATTCCAAGCA
IFNb	TCCCTATGGAGATGACGGAG	ACCCAGTGCTGGAGAAATTG
survivin	ATCCACTGCCCTACCGAGAA	CTTGGCTCTCTGTCTGTCCAGTT
CXCL10	CATCCCTGCGAGCCTATCC	CATCTCTGCTCATCATTCTTTTTCA
TNFα	ATGAGCACAGAAAGCATGA	AGTAGACAGAAGAGCGTGGT

Supplementary Figures

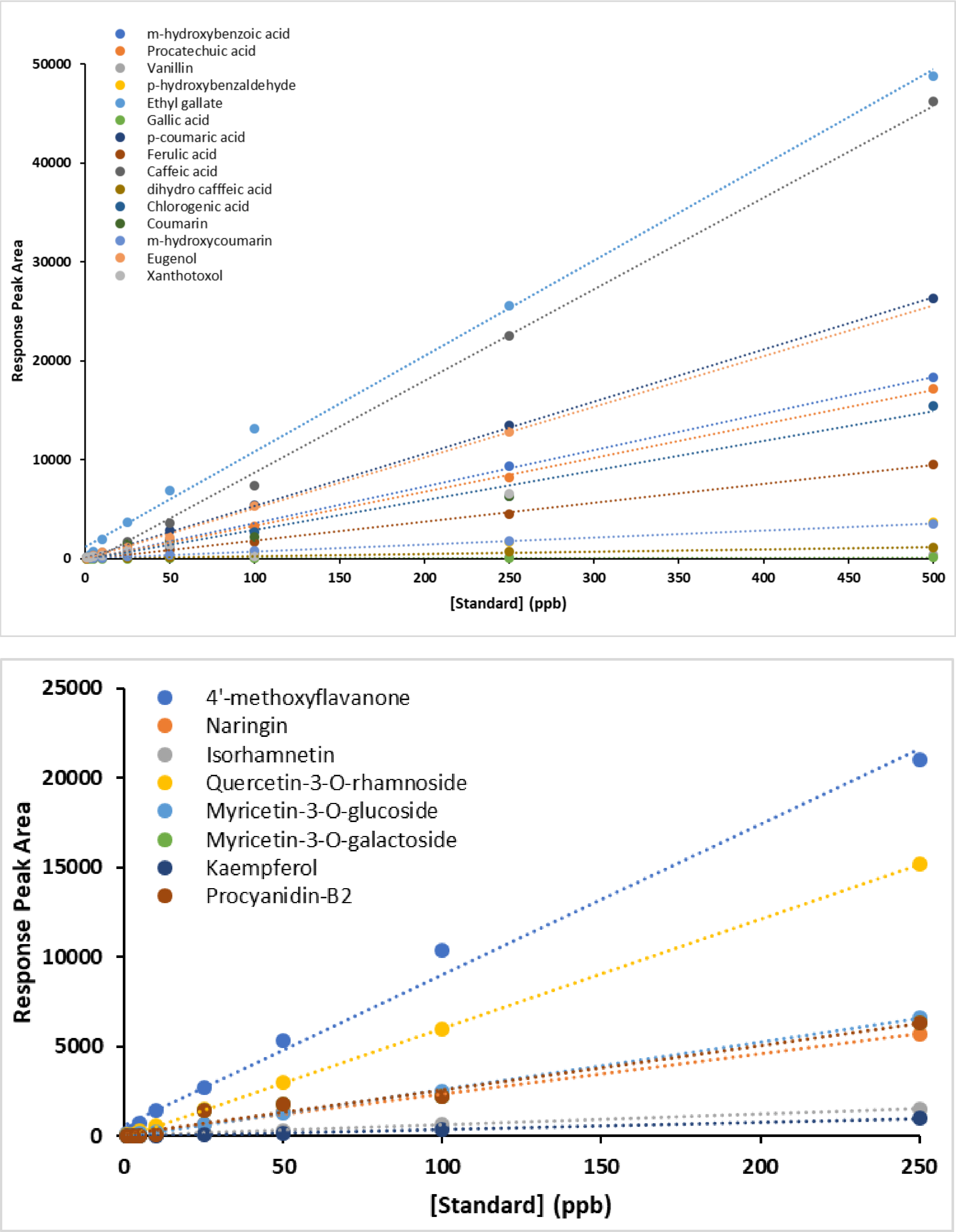

Figure S1: Calibration curve of polyphenolic acids in a range of concentrations (0-500 ppb for phenolic acids and 1.95-250 ppb for flavonoids).

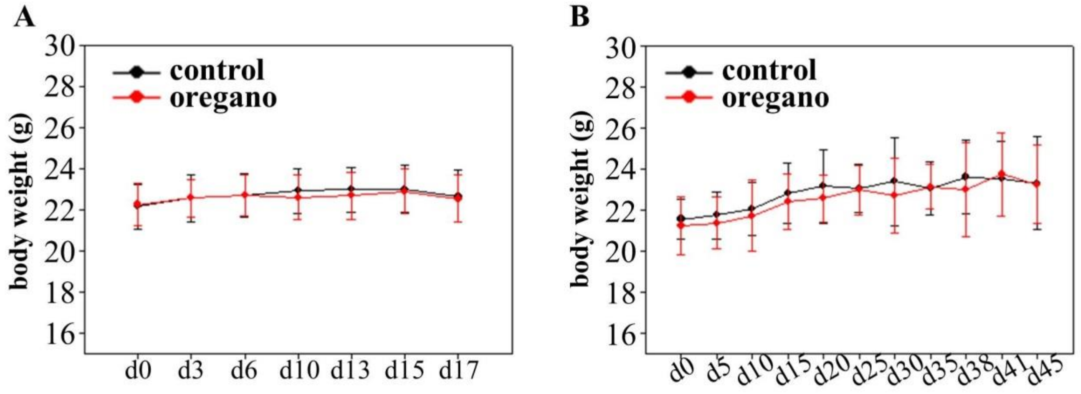

Figure S2: Animal body weight throughout the experiments. (A) Short term protocol of essential oil administration (0.348 mg/kg of body weight) dispersed in corn oil. Control mice were administered just corn oil. (B) Long term protocol of essential oil emulsion administration (350 ppm) diluted in tomato juice. Control group received tomato juice supplemented with the emulsion carrier but no essential oil. No significant differences were detected in animal body weight in either case.

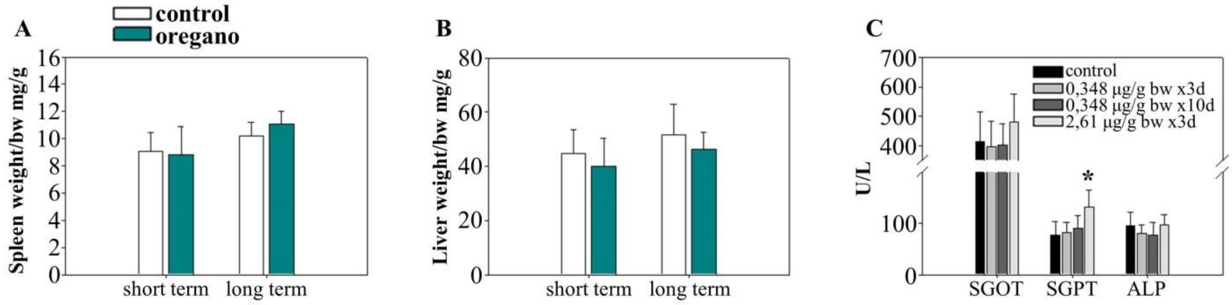

Figure S3: Evaluation of *Origanum* essential oil toxicity. There was no difference in the spleen or liver indices between tumor-bearing mice receiving the essential oil or not (A,B). We also measured serum liver enzymes of mice not harboring tumors that had received varying doses of the essential oil for 3 or 10 days (C). There was no difference in enzyme levels of animals receiving the experimental dose (0.348 mg/kg of bw) used in tumor experiments for either 3 or 10 days. Mice receiving 7.5 times more essential oil for 3 days had elevated levels of serum SGPT, but not SGOT or ALP.

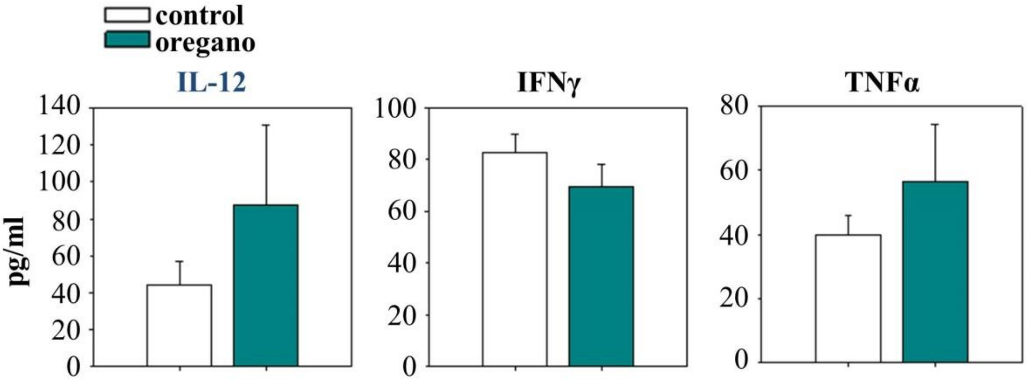

Figure S4: Cytokine concentration (IL-12, IFN-γ or TNF-α) in sera of control or oregano-treated mice in the short-term protocol. No significant differences were detected.

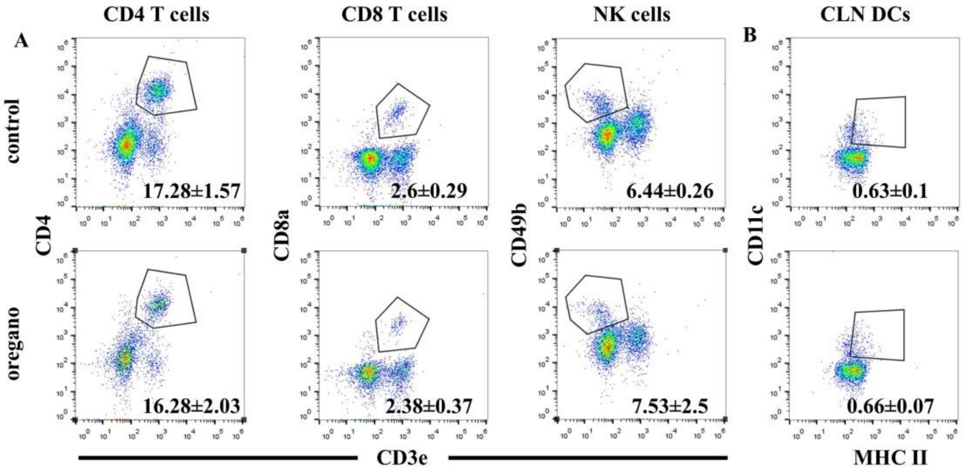

Figure S5: Immunophenotype analysis of tumor-infiltrating lymphocytes and cervical lymph node dendritic cells in the short-term experimental setting by flow cytometry. No significant accumulation or depletion of immune cells was evident in response to essential oil administration.

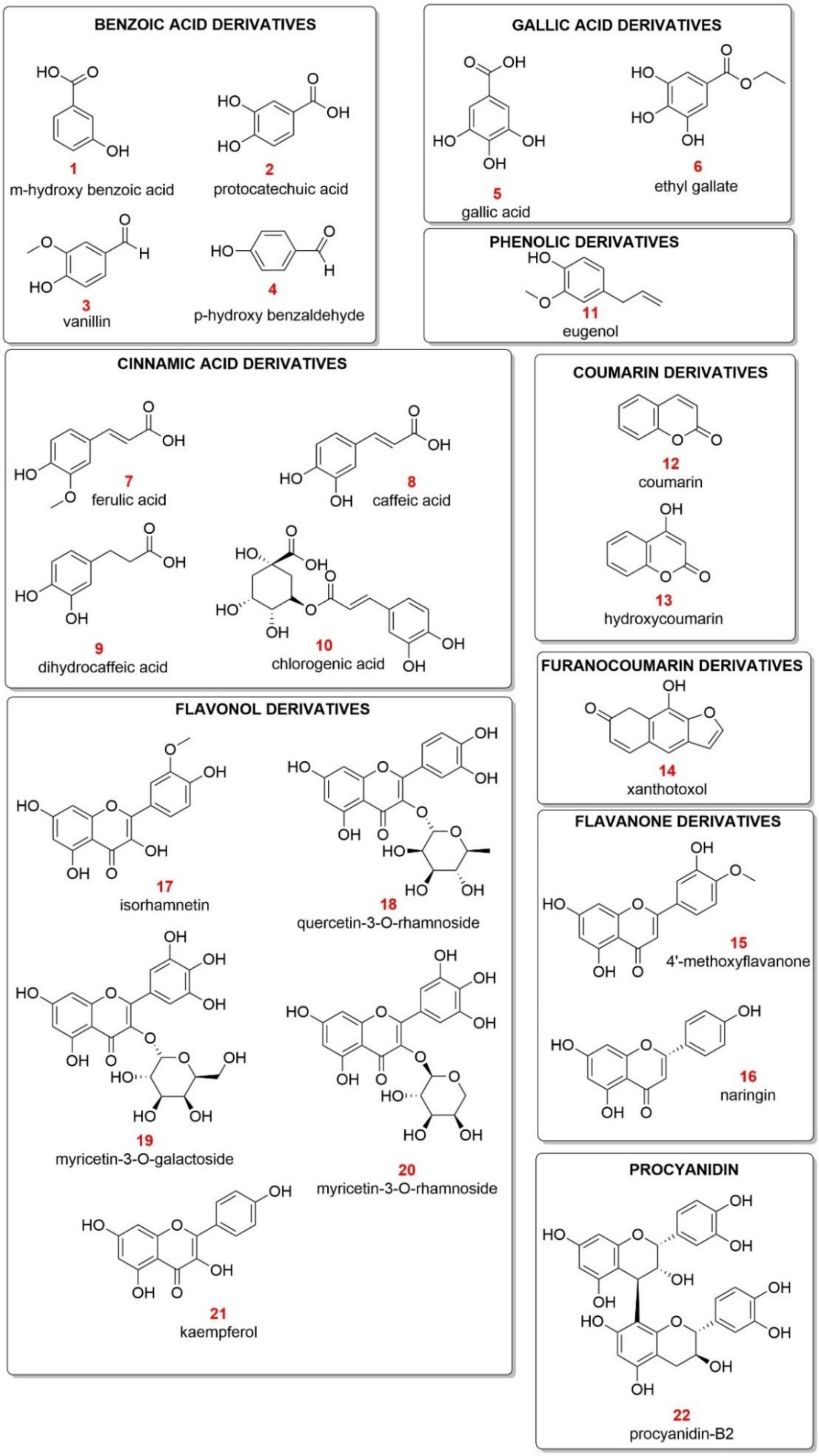

Figure S6: Structure of the identified molecules in *Origanum vulgare spp hirtum* oil.

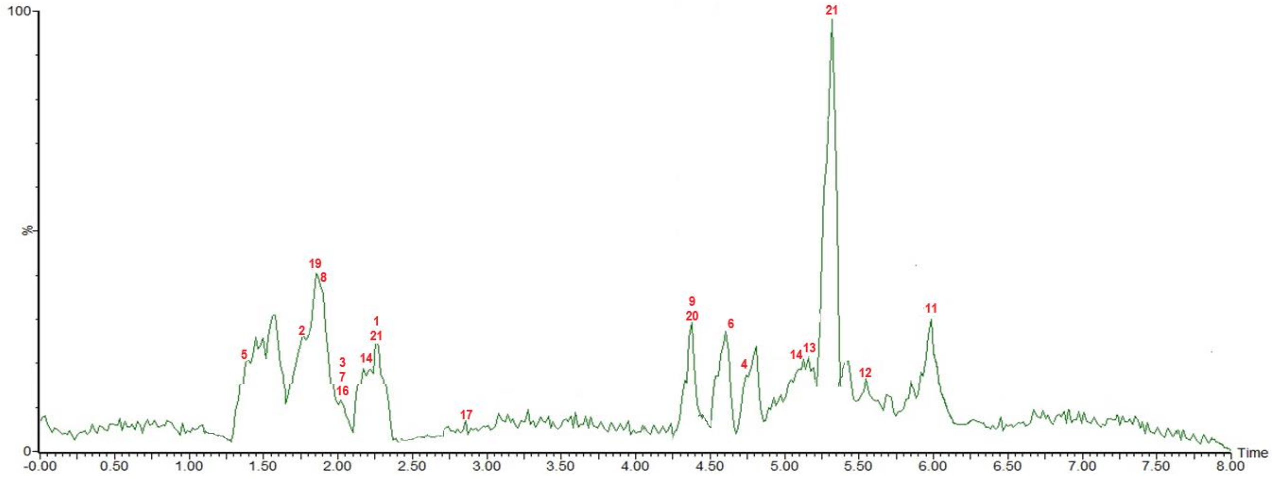

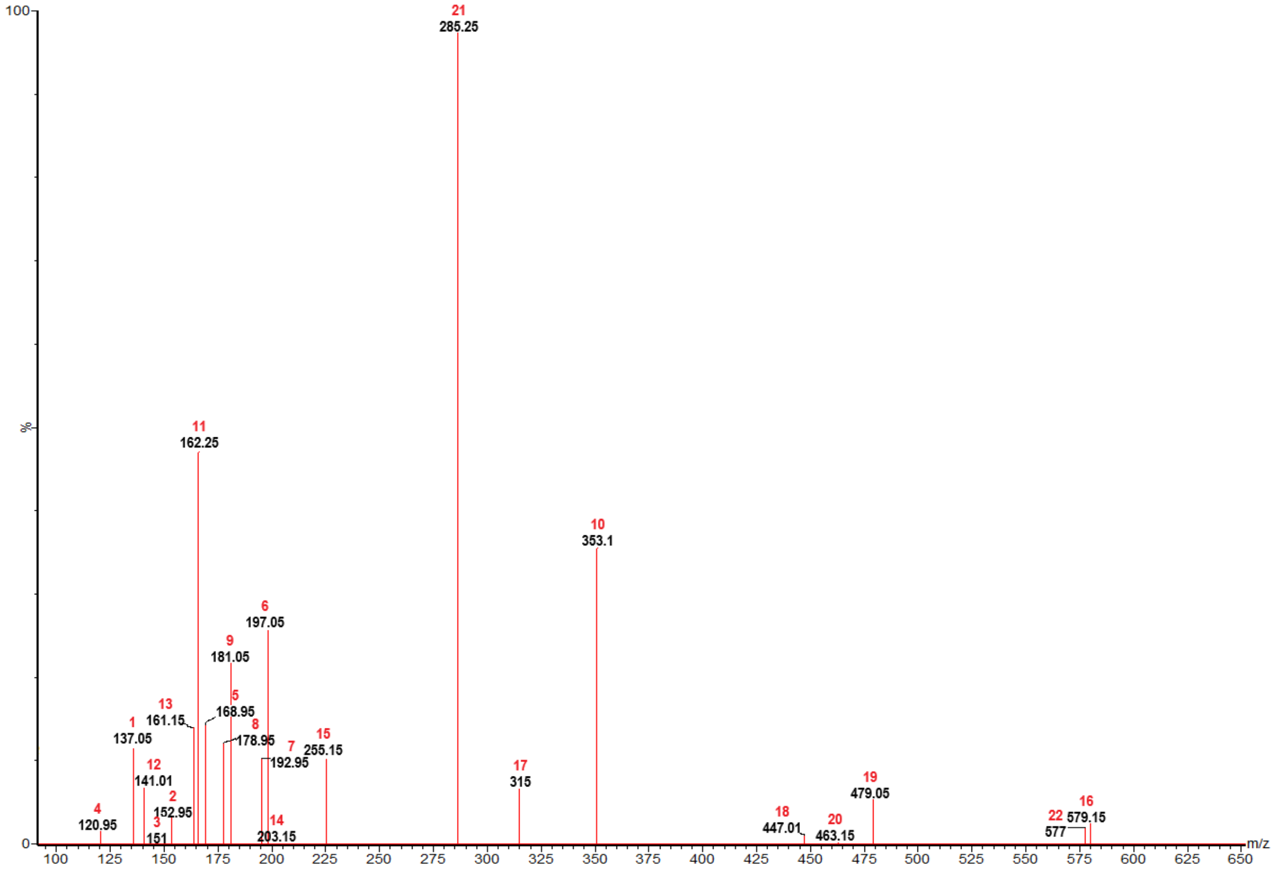

Figure S7: A) Chromatogram obtained upon UPLC-MS analysis of *Origanum vulgare spp hirtum* oil whereas B) shown the Selected Ion Recording (SIR) spectrum of the analytes.

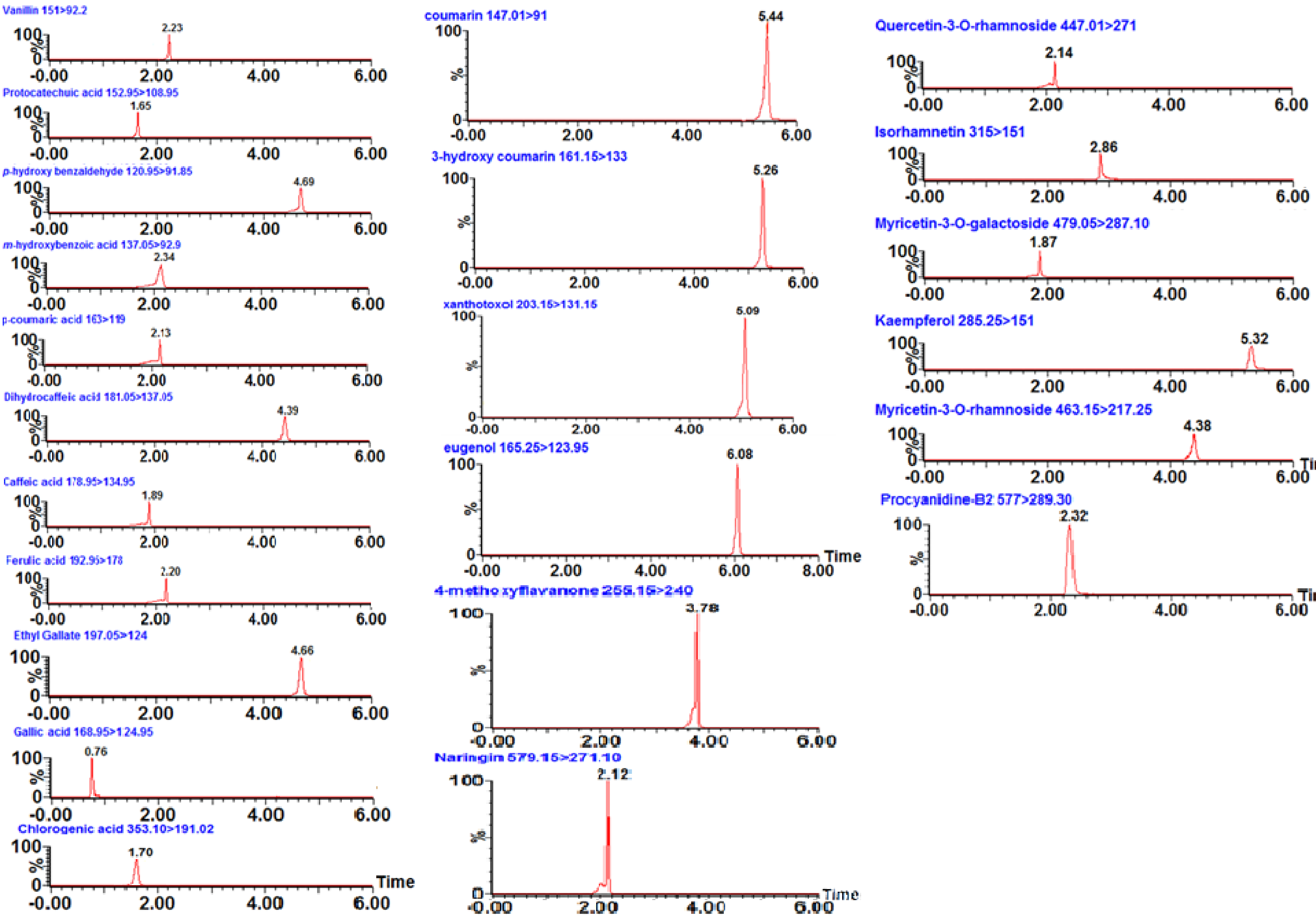

Figure S8: Extracted MS² chromatograms that were used for the targeted quantification of the isolated oil of *Origanum vulgare spp hirtum.*
